# Development and Validation of a Malay Version Questionnaire to Evaluate Remote Health Monitoring of COVID-19 via Telehealth Applications: Navigating Telehealth Evolution

**DOI:** 10.7759/cureus.67579

**Published:** 2024-08-23

**Authors:** Kalaivane Kannadasan, Maznah Dahlui, Farizah Mohd Hairi, Amirah Azzeri

**Affiliations:** 1 Department of Social and Preventive Medicine, Faculty of Medicine, Universiti Malaya, Kuala Lumpur, MYS; 2 Department of Research Development and Innovation, Universiti Malaya Medical Centre, Kuala Lumpur, MYS; 3 Department of Primary Care, Public Health Unit, Universiti Sains Islam Malaysia, Nilai, MYS

**Keywords:** questionnaire development and validation, patient satisfaction, digital health tools, psychometric validation, remote healthcare services, telehealth evaluation

## Abstract

Introduction

The rapid adoption of telehealth services due to the COVID-19 pandemic has highlighted the necessity for effective tools to evaluate patient experiences. This study developed and validated the Telehealth Usability, Acceptability, and Satisfaction Questionnaire (TUASQ) for virtual COVID-19 Assessment Centres (CACs) in Malaysia, aiming to comprehensively measure usability, acceptability, and satisfaction.

Methodology

The TUASQ was developed in two phases. Initially, the questionnaire development phase included item generation guided by the Technology Acceptance Model (TAM) and the Health Belief Model (HBM), with feedback from a panel of six experts. Items were refined through Content Validity Index (CVI) - Item-Level Content Validity Index (I-CVI) ≥ 0.82, Scale-Level Content Validity Index (S-CVI) ≥ 0.82, and Content Validity Ratio (CVR ≥ 0.78); and Face Validity Index (FVI) by 10 respondents - Item-Level Face Validity Index (I-FVI) ≥ 0.82 and Scale-Level Face Validity Index (S-FVI ≥ 0.82). The psychometric validation phase involved a cross-sectional study of 705 respondents, recruited through convenience sampling from March to July 2024, to perform Exploratory Factor Analysis (EFA) and Confirmatory Factor Analysis (CFA), followed by reliability testing using Cronbach's alpha, Composite Reliability (CR), and Average Variance Extracted (AVE).

Results

Content validation showed that most items' I-CVI exceeded 0.82, indicating significant expert consensus on relevance and clarity. The CVR also surpassed the 0.78 threshold, affirming their essential role. Face validation indices generally exceeded 0.80, confirming the questionnaire’s clarity and comprehensiveness from the users’ perspective. EFA with 250 participants indicated a high Kaiser-Meyer-Olkin Measure of Sampling Adequacy (KMO) of 0.933 and significant Bartlett’s test (χ² (136) = 3752.698, p < 0.001), supporting the factorability of the data and extracting three distinct factors. CFA with 455 participants initially showed a poor fit, prompting model adjustments that subsequently improved the fit indices (Root Mean Square Error of Approximation (RMSEA) = 0.076, Standardized Root Mean Square Residual (SRMR) = 0.045, Goodness of Fit Index (GFI) = 0.94, Tucker-Lewis Index (TLI) = 0.96, Comparative Fit Index (CFI) = 0.97). Reliability testing revealed a high internal consistency with Cronbach’s alpha of 0.975. CR for each factor exceeded the 0.70 threshold, and the AVE for each factor was above 0.50, indicating good convergent validity.

Conclusion

The validated TUASQ is a reliable and effective instrument for assessing the experiences of Malaysian patients using virtual CAC. Demonstrating robust psychometric properties through comprehensive validation processes, the TUASQ accurately measures usability, acceptability, and satisfaction, identifying strengths and areas for improvement in telehealth services. This contributes to enhanced care quality and patient satisfaction in the evolving healthcare landscape.

## Introduction

The COVID-19 pandemic has significantly accelerated the global adoption of telehealth services, reshaping the landscape of healthcare access and delivery. Telehealth, which involves the remote provision of healthcare services through digital and telecommunication technologies, has become a critical component of healthcare systems. This modality offers improved access to care, enhanced patient safety, and efficient management of patient care, especially during public health emergencies by minimizing physical interactions and reducing the transmission of infectious diseases [1,2].

In Malaysia, the introduction of virtual COVID-19 Assessment Centres (CACs) was a key response to the pandemic. These centres facilitate the remote monitoring of COVID-19 patients, allowing healthcare providers to assess and manage patient conditions without necessitating physical visits to healthcare facilities. This strategy not only mitigates the risk of virus transmission but also alleviates pressure on healthcare systems by conserving resources for those with severe symptoms [3].

Despite the swift integration of telehealth into mainstream healthcare, its success largely depends on patient acceptance. Understanding patient experiences and perceptions is crucial for evaluating the usability, acceptability, and satisfaction with telehealth services. This understanding helps identify barriers to usage, such as technological difficulties or privacy concerns, and informs strategies to enhance patient engagement and satisfaction [4,5].

The necessity for reliable and valid tools to assess patient experiences with telehealth services, such as virtual CACs, is increasingly apparent. Given the diversity in the design and functionality of telehealth platforms, a standardized method for evaluation ensures that feedback is consistent and actionable. Comprehensive evaluation tools enable the assessment of various aspects, including the ease of use of the technology, the clarity and relevance of the information provided, and the quality of communication between patients and healthcare providers [5-7].

The study aims to develop a questionnaire to address specific gaps found in other existing tools for assessing telehealth services. Tailored to the Malaysian healthcare context to capture the unique challenges of virtual patient monitoring and offers a comprehensive evaluation that includes usability, acceptability, and satisfaction. This makes the developed questionnaire a valuable tool for gaining a more complete understanding of patient experiences and for optimizing telehealth delivery in various healthcare settings. The development of a specialized questionnaire for assessing telehealth services is essential for several reasons: it provides a structured method to gather patient feedback, highlights areas of excellence such as the convenience of receiving healthcare services remotely without the need to travel and those needing improvement such as improvement in the area of communicating with healthcare workers (HCWs) through the virtual platform, and serves as a benchmark for comparing different telehealth applications, thus facilitating best practice sharing and setting industry standards [8,9].

This study details the development and validation of the Telehealth Usability, Acceptability, and Satisfaction Questionnaire (TUASQ), which aims to enhance the quality and effectiveness of telehealth services, ensuring they meet patient needs and improve healthcare delivery.

## Materials and methods

The methodology for developing and validating the TUASQ consisted of two main phases: Questionnaire development and psychometric validation.

Phase 1: Questionnaire development

The initial phase focused on the conceptualization and construction of the TUASQ, incorporating several key steps:

Item Generation

**Figure 1:**
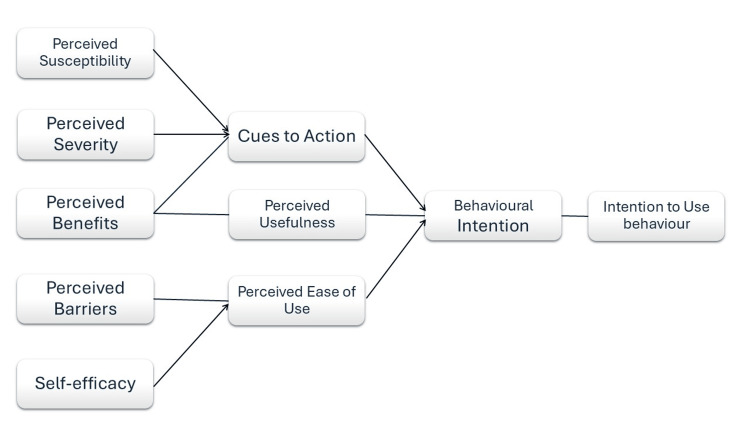
Integrated TAM and HBM model of theoretical framework TAM: Technology Acceptance Model; HBM: Health Belief Model

TUASQ was guided by an integrated approach using the Technology Acceptance Model (TAM) and the Health Belief Model (HBM). TAM contributed by emphasizing the importance of perceived usefulness and ease of use, which informed the creation of items that assess how these factors influence patient acceptance and usability of the telehealth platform. HBM added a health-focused perspective, highlighting the role of perceived severity, susceptibility, benefits, and barriers in patient engagement with telehealth services. By combining these models, TUASQ was able to incorporate both technological and health behavior aspects into its items and factors, ensuring a comprehensive evaluation of patient experiences with telehealth [10,11]. The item generation process also drew from established tools such as the Telehealth Usability Questionnaire (TUQ) and the Malay Version of the mHealth App Usability Questionnaire (m-MAUQ) to ensure cultural and linguistic relevance [9,12]. The questionnaire was designed to comprehensively cover three domains: Usability, Acceptability, and Satisfaction, comprising a total of 21 items [13]. Usability items assessed the ease of navigation, interface design, and clarity of information [14]. Acceptability items focused on the perceived usefulness and benefits of the telehealth service [15]. Satisfaction items gauged overall user satisfaction, including service quality and user experience [16]. A certified expert in the Malay language was recruited at this stage to refine the wording and ensure the clarity and appropriateness of the language used in the instrument. All items were measured using a five-point Likert scale, ranging from 1 (strongly disagree), 2 (disagree), 3 (neutral), 4 (agree) and 5 (strongly agree), allowing respondents to express varying degrees of agreement or satisfaction [17]. The integrated TAM and HBM model used for the TUASQ item generation is portrayed in Figure 1.

Content Validation

Referring to Yusoff [18], a panel of six experts, including two public health professionals, two telehealth practitioners, and two academic researchers, evaluated the generated items for relevance, clarity, and comprehensiveness.

For the Content Validity Index (CVI) calculation, the experts rated each item on a four-point Likert scale of 1 (not relevant), 2 (somewhat relevant), 3 (quite relevant) and 4 (highly relevant), assessing relevance, clarity, and necessity. The ratings were used to calculate the Item-Level Content Validity Index (I-CVI) and Scale-Level Content Validity Index (S-CVI). The I-CVI for each item was calculated as the proportion of experts rating the item as either 3 (quite relevant) or 4 (highly relevant). The formula for I-CVI is I-CVI = (number of experts who rated the item as 3 or 4)/(total number of experts). Items with I-CVI scores of 0.78 or higher were considered acceptable. Items scoring below this threshold were revised or discarded. As for SVI, the S-CVI/Ave (average of I-CVI values for all items) was calculated, with a target value of 0.80 or higher for the overall questionnaire, indicating excellent content validity.

Additionally, the Content Validity Ratio (CVR) was calculated for each item to assess the necessity of its inclusion. Each expert is asked to rate every item in the questionnaire based on its essentiality to the construct being measured. The experts use a three-point scale: 1 indicates the item is not essential, 2 suggests the item is useful but not essential, 3 means the item is essential and 4 indicates definitely essential. Experts rated each item on a scale where ratings of 1 and 2 indicated the item was "not essential," while ratings of 3 and 4 signified the item was "essential." After collecting the ratings, the CVR for each item is calculated using the formula: CVR = (n_e - N/2)/(N/2), where n_e is the number of experts who rated the item as "essential," and N is the total number of experts. The CVR value ranges from -1 to +1, with +1 indicating unanimous agreement among experts that the item is essential, 0 indicating a 50% agreement, and negative values indicating less than 50% agreement. A higher CVR score reflects stronger agreement among the experts about the item's importance. For a panel of six experts, the minimum acceptable CVR value is 0.99, though a CVR value of at least 0.78 is generally considered acceptable. Items that do not meet this threshold should be revised or removed to maintain the questionnaire's overall validity [18].

Face Validation

After content validation, the questionnaire underwent face validation. A sample of 10 HCWs who are the end-users of the telehealth services assessed the clarity, relevance, and ease of understanding of the items. The Face Validity Index (FVI) was assessed at both the Item-Level Face Validity Index (I-FVI) and the Scale-Level Face Validity Index (S-FVI). I-FVI is calculated for each item by determining the proportion of reviewers who rated the item on a four-point Likert scale. Items rated as 1 (not clear and understandable) and 2 (somewhat clear and understandable) indicated as "not clear" whereas items rated 3 (clear and understandable) and 4 (very clear and understandable) rated as "clear." Specifically, the I-FVI is calculated using the formula: I-FVI = (number of reviewers who rated the item as 3 or 4)/(total number of reviewers). An I-FVI score of 0.78 or higher indicates that the item is clear and comprehensible to the majority of reviewers, ensuring that the item is suitable for inclusion in the final version of the questionnaire. S-FVI is assessed in two ways: S-FVI/UA and S-FVI/Ave. The S-FVI/UA (universal agreement) calculates the proportion of items that achieved unanimous agreement among reviewers, meaning all reviewers rated the item as clear or very clear. The S-FVI/Ave (average) represents the average of the I-FVI values across all items, providing an overall measure of the face validity of the entire scale. An S-FVI/Ave of 0.78 or higher is considered acceptable, indicating that the scale, on average, has good face validity [19].

Phase 2: Psychometric validation of TUASQ

A cross-sectional validation study was conducted to evaluate the psychometric properties of the TUASQ.

Participants

The study included Malaysian citizens who were 18 years or older, could converse in the Malay language, and consented to participate. Participants were classified as COVID-19 category 1 (asymptomatic) or category 2a (mild symptoms without pneumonia). Exclusion criteria included non-primary users of the telehealth application and respondents who were escalated to COVID-19 category 2b and above during the remote home monitoring period.

Sample Size Determination

Exploratory Factor Analysis (EFA) is used to identify the underlying factor structure of a set of variables. A commonly recommended guideline for determining the sample size for EFA is to have at least 5 to 10 participants per item, with a minimum of 100 to 200 participants to ensure stable factor solutions. In this study, the sample size for EFA was determined based on an estimated ratio of 10 respondents per item ensuring that it met these guidelines. There are 21 items in the questionnaire; hence, the minimum required sample size is 210 respondents. As for Confirmatory Factor Analysis (CFA), the participant-to-parameter ratio is crucial for determining an adequate sample size, typically recommended to be between 5:1 and 10:1 [20]. Followed by the quality of the sample size is categorized as follows: 100 participants is poor, 200 is fair and the minimum acceptable, 300 is good, 500 is very good, and 1000 or more is excellent. Hence for 21 items, taking into consideration of 10:1 ratio, 210 respondents are required. In total EFA and CFA, required 420 respondents, and considering a 20% nonresponsive rate, the sample size was inflated to 504 respondents [20].

Data Collection

Data collection occurred from March 2024 to July 2024 through the SELANGKAH telehealth application, which was utilized as a virtual CAC for remote home monitoring of COVID-19 patients. Convenience sampling was employed to recruit the respondents.

Measures

The research questionnaire included sociodemographic characteristics (age, gender, ethnicity, formal education status, and household income) and clinical characteristics relevant to telehealth service usage. The TUASQ contained 21 items across three domains: Usability (five items), Acceptability (10 items), and Satisfaction (six items). The items were presented using a five-point Likert scale, ranging from 1 (strongly disagree) to 5 (strongly agree).

Data Analysis

The second phase of the study focused on the empirical testing and validation of the TUASQ to establish its psychometric properties, ensuring both reliability and validity. The EFA aimed to identify the underlying factor structure of the TUASQ, utilizing data from a sample of COVID-19 patients who had used telehealth services. EFA was conducted using Statistical Package for the Social Sciences (IBM SPSS Statistics for Windows, IBM Corp., Version 29.0, Armonk, NY), where the Kaiser-Meyer-Olkin Measure of Sampling Adequacy (KMO) and Bartlett's test of sphericity assessed the data's suitability for factor analysis. A KMO value greater than 0.6 is generally acceptable, indicating adequate sampling adequacy, while a significant Bartlett’s test (p-value < 0.05) confirms sufficient correlations between items for EFA [21]. Factors were retained based on eigenvalues greater than 1, and items with factor loadings below 0.32 were considered for removal to better represent the factors. Following the EFA, CFA was performed to confirm the factor structure identified earlier. This analysis was executed using Stata/MP (version 17; StataCorp LLC, College Station, Texas, USA), applying several fit indices such as the Comparative Fit Index (CFI), Tucker-Lewis Index (TLI), Standardized Root Mean Square Residual (SRMR) and Root Mean Square Error of Approximation (RMSEA). The model was deemed to have a good fit if the CFI and TLI values exceeded 0.90, indicating an excellent fit between the data and the model, and an RMSEA and SRMR value below 0.08, suggesting reasonable error approximation [21]. CFA was pivotal in establishing the construct validity of the TUASQ by verifying the alignment of the items with their respective theoretical constructs. Lastly, the internal consistency of the questionnaire was evaluated through reliability testing for each factor identified. This was accomplished by calculating Cronbach's alpha, with values of 0.70 or higher deemed acceptable, indicating reliable internal consistency, while values of 0.80 or higher were considered good. Additionally, Composite Reliability (CR) and Average Variance Extracted (AVE) were computed to further assess the consistency and convergent validity of the constructs. CR values above 0.60 were indicative of good internal consistency, and AVE values greater than 0.50 suggested sufficient convergent validity, confirming a significant amount of variance in the indicators is captured by the construct [22,23].

Ethical considerations

The study was approved by the Malaysian Research Ethical Committee (MREC) of the Ministry of Health and was registered in August 2023 with the National Medical Research Registry (NMRR) under the approval number NMRR ID-22-01180-LQL. The study provided a Participant Information Sheet (PIS) detailing the study's objectives and procedures. Consent was obtained from all participants, ensuring their voluntary participation. Data confidentiality was maintained throughout the research process.

## Results

Phase 1

In content validation, the CVI was employed to evaluate the relevance of the questionnaire items. Most items had an I-CVI above 0.78, indicating high relevance. However, two items, Q5 and Q15, had I-CVI values of 0.7 and were subsequently removed. The CVR was also assessed, with the majority of items meeting the minimum threshold of 0.78, demonstrating the questionnaire's reliability. After revisions based on expert feedback, 19 items were retained.

The face validation of the 19-item TUASQ involved assessing clarity and comprehension with a group of 10 healthcare staff. The I-FVI for most items was 1.0, indicating perfect agreement among raters. A few items, including Q6, Q16, and Q20, showed slightly lower levels of agreement, with I-FVI values of 0.9 for Q6 and 0.80 for both Q16 and Q20. The overall scale-level face validity indices were S-FVI/Ave = 0.96 and S-FVI/UA = 0.84, indicating strong overall clarity and relevance across the questionnaire. No items were removed, and no further major modifications were required, confirming the items' clarity and comprehensibility.

Phase 2

The recruitment process for the study began by targeting a pool of COVID-19 patients from the Hulu Langat COVID-19 Registry, which included 220,848 individuals. From this large pool, the focus was narrowed to users of the SELANGKAH platform within the Hulu Langat district. After applying the inclusion and exclusion criteria, the pool was further refined to 8,868 eligible users. To recruit participants, convenience sampling was employed, resulting in a final sample of 705 respondents. These participants were then asked to provide demographic data, which included information on their gender, age, occupation, ethnicity, household income, and education level.

The sample included a majority of females (73.2%), with 516 women compared to 189 men (26.8%), with a mean age of 41.1 (SD = 8.9). The participants were employed across various sectors, with 32.9% working in the government sector, 26.0% in the private sector, 23.3% being unemployed or students, 15.9% self-employed, and 2.0% retired or pensioners. Ethnically, the study included 67.9% Malays, 23.0% Indians, 5.7% Chinese, and 3.4% from other ethnic groups.

In terms of household income, the majority of participants (88.8%) reported earning less than RM2209, while 8.2% earned between RM2210 and RM4849, 0.6% earned between RM4850 and RM10959, and 3.4% earned more than RM10960. Regarding education levels, 45.8% had secondary education, 41.0% had primary education, 9.6% had tertiary education, and 3.5% had no formal education (Table 1).

**Table 1:** Sociodemographic characteristics RM: Ringgit Malaysia

Characteristics		N	%
Age, years (mean age)		41.1 (SD = 8.9)	
Gender	Male	189	26.8
	Female	516	73.2
Occupation	Government	232	32.9
	Private Sector	183	26.0
	Self-employed	112	15.9
	Pensioner	14	2.0
	Unemployed/Students	164	23.3
Ethnicity	Malay	479	67.9
	Chinese	40	5.7
	Indian	162	23.0
	Others	24	3.4
Household income	less than RM2209	626	88.8
	RM2210-RM4849	58	8.2
	RM4850-RM10959	4	0.6
	more than RM10960	17	2.4
Education level	Primary	289	41.0
	Secondary	323	45.8
	Tertiary	68	9.6
	Unschooled	25	3.5

Factor analysis

**Figure 2:**
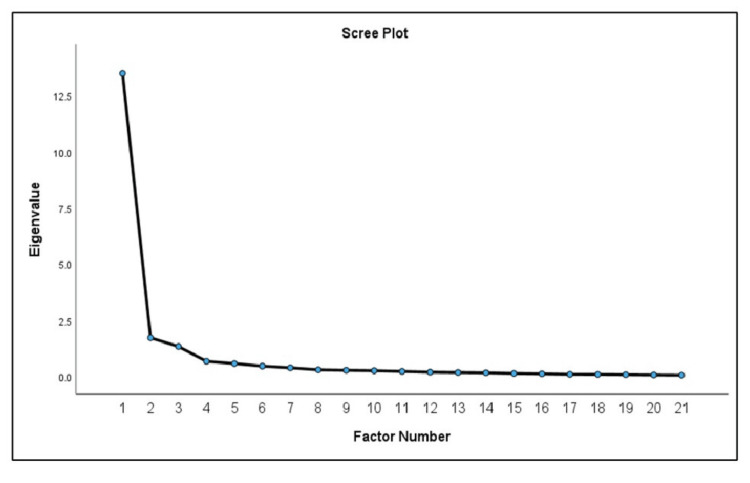
Scree plot of three factors extracted from principal axis factoring and Promax rotation

EFA was conducted with 250 respondents, confirming the adequacy of the sample size with a KMO value of 0.933, which indicates excellent sampling adequacy. Bartlett’s test of sphericity was highly significant (χ² (136) = 3752.698, p < 0.001), supporting the appropriateness of the factor analysis for the data. The item factor loadings, as shown in Table 2, ranged from 0.538 to 1.022, with no items falling below 0.32. Three factors were extracted based on the eigenvalues (Table 3) and the scree plot (Figure 2), with each factor having an eigenvalue greater than 1.

**Table 2:** Correlation matrix with factor loading values and communalities values of the 17-item TUASQ using Exploratory Factor Analysis SELANGKAH: Telehealth application used as virtual COVID-19 Assessment Centre for remote home monitoring of COVID-19 patients; TUASQ: Telehealth Usability, Acceptability, and Satisfaction Questionnaire

Items	Questions	Factor 1	Factor 2	Factor 3	Communalities
Q18	I feel comfortable communicating with the clinician using the SELANGKAH.	.998	-	-	.860
Q19	SELANGKAH is an acceptable way to receive healthcare services	.968	-	-	.824
Q20	I think the visits provided over the SELANGKAH system are the same as in-person visits	.919	-	-	.813
Q21	I am satisfied with the SELANGKAH application for home monitoring of COVID-19	.807	-	-	.732
Q10	It was simple and easy to learn to use SELANGKAH	.759	-	-	.720
Q11	I could understand the command given through SELANGKAH.	.755	-	-	.709
Q13	I would use SELANGKAH or any equivalent telehealth system again for healthcare service	.726	-	-	.638
Q14	By using SELANGKAH I could get in touch with the healthcare personnel as well as if we met in person	.683	-	-	.706
Q16	Whenever I made a mistake in SELANGKAH, I could rectify it easily and quickly	.683	-	-	.606
Q8	I like using SELANGKAH.	-	.935	-	.859
Q9	SELANGKAH can do everything I would want it to be able to do.	-	.916	-	.887
Q4	It was simple to use this SELANGKAH application	-	.891	-	.877
Q6	I believe I could become productive quickly using this SELANGKAH system	-	.798	-	.713
Q7	The way I interact with this SELANGKAH is pleasant.	-	.710	-	.707
Q1	SELANGKAH improves my access to healthcare services for COVID-19 monitoring.	-	-	1.022	.985
Q2	SELANGKAH saves me time traveling to a COVID-19 Assessment and Treatment Centre.	-	-	.725	.579
Q3	SELANGKAH provides for my healthcare needs (e.g. consultation, treatment, counselling, medication delivery, etc.)	-	-	.538	.543

**Table 3:** Eigenvalues and total variance values of TUASQ Extraction Method: Principal Axis Factoring. ^a ^When factors are correlated, sums of squared loadings cannot be added to obtain a total variance; TUASQ: Telehealth Usability, Acceptability, and Satisfaction Questionnaire

Factor	Initial Eigenvalues			Extraction Sums of Squared Loadings				Rotation Sums of Squared Loadings^a^
	Total	% of Variance	Cumulative %		Total	% of Variance	Cumulative %	Total
1	13.489	64.233	64.233	13.253		63.111	63.111	11.817
2	1.731	8.245	72.478	1.507		7.176	70.287	11.208
3	1.338	6.372	78.850	1.095		5.214	75.500	5.793
4	.682	3.246	82.096	-		-	-	-
5	.581	2.768	84.864	-		-	-	-
6	.466	2.220	87.084	-		-	-	-
7	.392	1.869	88.953	-		-	-	-
8	.304	1.445	90.398	-		-	-	-
9	.281	1.340	91.739	-		-	-	-
10	.263	1.254	92.993	-		-	-	-
11	.231	1.100	94.094	-		-	-	-
12	.198	.944	95.038	-		-	-	-
13	.183	.872	95.910	-		-	-	-
14	.170	.809	96.719	-		-	-	-
15	.141	.671	97.390	-		-	-	-
16	.125	.593	97.984	-		-	-	-
17	.101	.482	98.465	-		-	-	-
18	.097	.464	98.930	-		-	-	-
19	.087	.414	99.344	-		-	-	-
20	.074	.354	99.697	-		-	-	-
21	.064	.303	100.000	-		-	-	-

These factors were further refined using Principal Axis Factoring (PAF) with promax rotation [23]. While no items had factor loadings less than 0.3, cross-loadings were identified between items Q11-Q14 and items Q9-Q10 for Factors 1 and 2. To resolve these cross-loadings, items with low communality values were systematically removed. This process led to the removal of items Q9 and Q11, resulting in a refined 17-item version of the TUASQ.

EFA extracted 17 items under three main domains: Acceptability, Satisfaction, and Usability, each aligning with relevant theoretical frameworks.

Factor 1: Acceptability

Nine items were grouped under the domain of Acceptability, reflecting aspects such as learnability, comprehensibility, future use intention, communication, error handling, comfort, practicality, equivalence to face-to-face interactions, and engagement. These items assess how easily users can learn and understand the telehealth system, their willingness to use it in the future, their comfort and satisfaction with communication and error handling, and how well the system meets their healthcare needs compared to traditional face-to-face services.

Factor 2: Satisfaction

Five items were categorized under Satisfaction, focusing on friendliness, ease of use, productivity, comprehensibility, and overall satisfaction with the system. These items gauge how user-friendly and productive the SELANGKAH system is, how well users understand the provided instructions, and their overall contentment with the system's performance in fulfilling their healthcare needs.

Factor 3: Usability

Three items were identified under the Usability domain, emphasizing efficiency, ease of use, and effectiveness. These items assess how the telehealth system saves time by eliminating the need for transportation to healthcare centers, its user-friendliness in home-based monitoring, and its effectiveness in providing comprehensive healthcare services, including medical consultations, counseling, and medication delivery.

**Figure 3:**
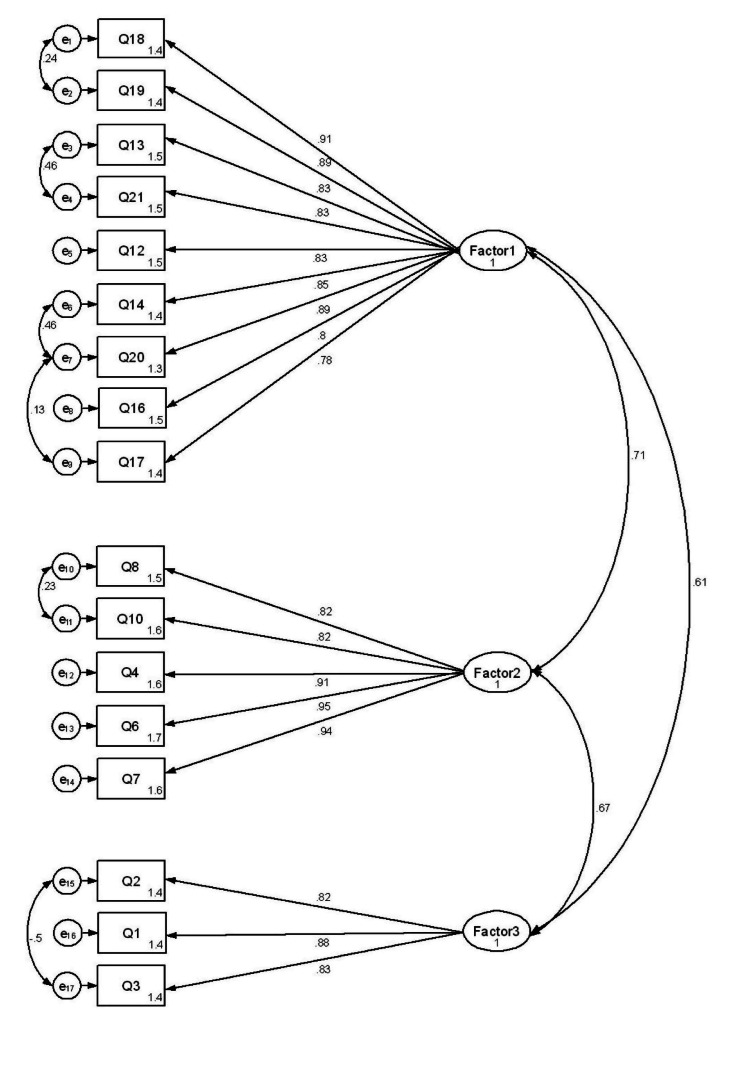
The 17-item TUASQ measurement model from Confirmatory Factor Analysis Each item is represented by a box, and each factor is represented by a circle. The arrows from the circle (factor) to the box (item) indicate the factor loadings. e represents measurement errors in each item. TUASQ: Telehealth Usability, Acceptability, and Satisfaction Questionnaire

Following EFA, CFA was conducted using Structural Equation Modelling (SEM) with data from 455 respondents. The CFA model was initially constructed with three factors and 17 items, where all factor loadings were above 0.6 as shown in Figure 3.

However, the initial model did not fit well, as indicated by the goodness-of-fit indices: RMSEA = 0.123, SRMR = 0.058, Goodness of Fit Index (GFI) = 0.94, TLI = 0.89, and CFI = 0.90. To improve the model fit, 11 items were adjusted as free parameter estimates in pairs, based on high maximum likelihood values. This adjustment resulted in standardized factor loadings ranging from 0.78 to 0.95, indicating strong associations between the items and their respective constructs. The final model demonstrated a good fit, with improved indices: RMSEA = 0.076, SRMR = 0.045, GFI = 0.94, TLI = 0.96, and CFI = 0.97. These results indicate that the refined model accurately captured the constructs measured by the TUASQ (Table 4).

**Table 4:** Goodness of Fit indices values of 17 item-TUASQ TUASQ: Telehealth Usability, Acceptability, and Satisfaction Questionnaire; RMSEA: Root Mean Square Error of Approximation; SRMR: Standardized Root Mean Square Residual; GFI: Goodness of Fit Index; TLI: Tucker-Lewis Index; CFI: Comparative Fit Index

Category of Fit	Index Name	Acceptance Level	Output	Description
Absolute Fit Index	RMSEA	<0.08	0.076	Fit
	SRMR	<0.08	0.045	Fit
Incremental Fit Indices	GFI	>0.9	0.94	Fit
	TLI	>0.95	0.96	Fit
	CFI	>0.95	0.97	Fit

Reliability

The questionnaire exhibited a high level of internal consistency, with an overall Cronbach's alpha of 0.975, indicating excellent reliability. The CR values were consistently strong across the factors, each above 0.75, demonstrating that the items reliably measure their respective constructs. The AVE values were also significant, all exceeding 0.70, which indicates that the constructs account for a significant portion of the variance in the items. These overall results confirm that the TUASQ is a highly reliable and valid instrument for assessing the dimensions of acceptability, satisfaction, and usability in telehealth services (Table 5).

**Table 5:** Factor Loadings, Composite Reliability (CR), Average Variance Extracted (AVE), and Cronbach's Alpha values of 17-item TUASQ TUASQ: Telehealth Usability, Acceptability, and Satisfaction Questionnaire

Factor	Item	Factor Loading	CR	AVE	Cronbach's Alpha
Acceptability	Q18	0.914	0.843	0.710	0.975
	Q19	0.864			
	Q13	0.833			
	Q21	0.832			
	Q12	0.816			
	Q14	0.852			
	Q20	0.919			
	Q16	0.782			
	Q17	0.759			
Satisfaction	Q8	0.823	0.888	0.789	0.973
	Q10	0.829			
	Q4	0.917			
	Q6	0.951			
	Q7	0.935			
Usability	Q2	0.822	0.847	0.718	0.976
	Q1	0.884			
	Q3	0.834			

## Discussion

The study aimed to develop and validate a questionnaire in Malay that assesses patient experiences in terms of acceptability, usability, and satisfaction with telehealth services used for remote home monitoring during the COVID-19 pandemic. In the initial phase, the questionnaire was constructed based on an extensive literature review and guided by the TAM and HBM, with particular emphasis on the m-MAUQ tool by Mustafa et al. [12]. This framework led to the formation of three key domains - Acceptability, Usability, and Satisfaction - comprising a total of 21 items: 10 items for acceptability, five items for usability, and six items for satisfaction. Following content and face validation, 19 items were retained, and ultimately, the questionnaire was finalized with 17 items after conducting EFA and CFA with three items in Usability, nine items in Acceptability, and five in Satisfaction domains.

Six-panel experts were meticulously selected based on their extensive experience and expertise in relevant fields. Among them were two public health specialists with strong knowledge of infectious diseases, including COVID-19, and experience as commanding officers of the virtual CACs in Hulu Langat district. Additionally, two telehealth practitioners were recruited for their familiarity with the telehealth platform and its application in monitoring patients remotely. Additionally, two academic experts, well-versed in health promotion and the HBM, were brought on board to offer guidance on the instrument's items, particularly concerning the domains identified through factor analysis. A certified Malay language expert was also recruited to ensure the linguistic clarity and appropriateness of the wording in the questionnaire.

To achieve consensus among the experts, CVI and CVR were employed, supplemented by qualitative feedback that further enhanced the relevance and clarity of the items. Following this, face validation was conducted with HCWs, who were also end-users of the telehealth platform. These HCWs, having been monitored remotely through the platform, provided critical insights based on their professional experience in patient care and their practical use of the telehealth application.

Involving HCWs in the face validation process significantly enriched the study by leveraging their strong understanding of both the medical and technical aspects of the telehealth system. Their feedback was crucial in identifying and resolving any ambiguities or issues within the questionnaire, thereby enhancing the accuracy of the data collected. This rigorous vetting process ensured that the final questionnaire effectively captured the intended constructs of acceptability, usability, and satisfaction. The FVI results demonstrated a high level of clarity and comprehensibility across all three domains, indicating a robust response process and confirming the questionnaire's efficacy.

Following, construct validation in this study was conducted using EFA and CFA. EFA validated the domains identified through the literature review by extracting three factors - Acceptability, Usability, and Satisfaction - and aligning the respective items with each factor. The KMO measure indicated excellent sampling adequacy, supporting the suitability of the data for factor analysis. CFA further validated the factor structure, with GFIs demonstrating an excellent model fit. To achieve this, two items were removed due to cross-loading, which improved the model's fit. The finalized TUASQ consists of three factors and 17 items: Acceptability (nine items), Usability (three items), and Satisfaction (five items).

The TUASQ demonstrated significant psychometric properties, including high internal consistency and validity. The study conducted reliability testing using multiple measures to ensure the robustness of the questionnaire. Convergent and Discriminant Validity were assessed through the AVE, which indicates that the items accurately represent their intended constructs and are distinct from other constructs, confirming the validity of the model. CR was also calculated demonstrating consistent internal measurements within each construct. Additionally, Cronbach’s alpha was employed indicating that the questionnaire items are reliable and effectively measure the same construct. The high AVE, CR, and Cronbach's alpha values across all domains indicate the reliability of the TUASQ in measuring these constructs, making it a valuable tool for healthcare providers and researchers [24,25].

The development of the TUASQ marks a significant advancement in evaluating patient experiences with telehealth services. The COVID-19 pandemic has highlighted the critical role of telehealth in providing healthcare access while minimizing physical interactions. As telehealth becomes increasingly integrated into mainstream healthcare, understanding patient experiences is essential for the continuous improvement of these services [26]. Hence, TUASQ serves as a crucial tool for systematically capturing patient feedback on telehealth services. This feedback is vital for identifying strengths and pinpointing areas that require enhancement. The relevance of this questionnaire extends beyond general patient experience assessment, as it provides specific insights into usability, acceptability, and satisfaction, all of which are critical for the effective deployment of telehealth services [27].

Importantly, this study represents one of the few efforts, to the best of our knowledge, to develop a questionnaire in Malay, specifically designed for the Malaysian cultural context. This cultural and linguistic adaptation ensures that the TUASQ accurately captures the nuances of patient experiences in Malaysia, making the findings highly relevant and actionable for local healthcare providers and policymakers. The development of the TUASQ not only fills a gap in the existing literature but also serves as a model for other regions looking to create culturally sensitive evaluation tools for telehealth services [28].

The study has several limitations that must be acknowledged. The use of convenience sampling may limit the generalizability of the findings, as the sample may not be fully representative of the broader population. Additionally, the study was conducted in the specific context of the COVID-19 pandemic, which may influence patient experiences differently than in normal circumstances. Another notable limitation is the questionnaire's development in Malay, which, while culturally relevant for the Malaysian population, may require linguistic and cultural adaptations for use in other language-speaking populations. Such adaptations should be accompanied by thorough validation processes to ensure the reliability and validity of the questionnaire in different contexts [29].

Future research should focus on validating the TUASQ across diverse populations and in various healthcare settings to enhance its generalizability. Additionally, as telehealth technologies continue to evolve, it is crucial to regularly update and validate the TUASQ to maintain its relevance and effectiveness. Future studies could also explore the impact of advanced technological features, such as artificial intelligence and machine learning, on patient experiences in telehealth settings [30].

## Conclusions

The TUASQ is a significant contribution to the field of telehealth research, providing a reliable and culturally relevant tool for assessing patient experiences in Malaysia. The insights gained from this study underscore the importance of patient-centered approaches in the design and implementation of telehealth services. As telehealth continues to expand, tools like the TUASQ will be indispensable for ensuring that these services meet the evolving needs and expectations of diverse patient populations, ultimately enhancing the quality and effectiveness of healthcare delivery.

## Appendices

Telehealth Usability, Acceptability and Satisfaction Questionnaire (TUASQ)

Dear Respondents,

Thank you for participating in this study. Your responses are essential in helping us evaluate the usability, acceptability, and satisfaction with the SELANGKAH Telehealth application, which has been used as a virtual COVID-19 Assessment Centre for remote home monitoring.

Instructions:

Section A: Sociodemographic Information

Please provide accurate details about your age, gender, occupation, ethnicity, household income, and education level by selecting the appropriate options. Please tick (✓) the boxes that apply to you.

*Section B: Perception on SELANGKAH* *Telehealth Application as virtual COVID-19 Assessment Centre*

Read each statement carefully and select the response that best reflects your experience with the SELANGKAH application. Use the provided scale ranging from "Strongly Disagree" to "Strongly Agree." Please tick (✓) the box corresponding to your answer.

Your responses are anonymous and will only be used for research purposes. We appreciate your honest feedback. Your honest feedback is highly valued and will contribute to the improvement of telehealth services.

Thank you for your time and cooperation.

SECTION A: Sociodemographic

Age                                        : ___________________________ (years)

Gender                                  :☐Male

                                               ☐Female

Occupation                           :☐ Government

                                               ☐ Private Sector

                                               ☐Self-Employed 

                                               ☐Pensioner

                                               ☐Unemployed/Student

Ethnicity                               :☐Malay

                                              ☐Chinese

                                              ☐Indian

                                              ☐Others

Household Income             :☐ less than RM2209

                                             ☐RM 2210 - RM 4849

                                             ☐ RM 4850 - RM 10 959

                                             ☐ More than RM 10 960

Education Level                 :☐Primary education

                                             ☐Secondary education

                                             ☐Tertiary education

                                             ☐No formal education

*SECTION B: Perception on SELANGKAH* *Telehealth application as virtual COVID-19 Assessment Centre*

**Table 6:** Questions on user experience with the SELANGKAH telehealth application as virtual COVID-19 Assessment Centre

Questions		Strongly Disagree	Disagree	Neutral	Agree	Strongly Disagree
Q1	SELANGKAH improves my access to healthcare services for COVID-19 monitoring.					
Q2	SELANGKAH saves me time traveling to a COVID-19 Assessment and Treatment Centre.					
Q3	SELANGKAH provides for my healthcare needs (e.g. consultation, treatment, counselling, medication delivery, etc.)					
Q4	It was simple to use this SELANGKAH application					
Q5	SELANGKAH is simple and easy to understand.					
Q6	I believe I could become productive quickly using this SELANGKAH system					
Q7	The way I interact with this SELANGKAH is pleasant.					
Q8	I like using SELANGKAH.					
Q9	SELANGKAH can do everything I would want it to be able to do.					
Q10	It was simple and easy to learn to use SELANGKAH					
Q11	I could understand the command given through SELANGKAH.					
Q12	I could easily communicate to the healthcare personnel using SELANGKAH.					
Q13	I would use SELANGKAH or any equivalent telehealth system again for healthcare service					
Q14	By using SELANGKAH I could get in touch with the healthcare personnel as well as if we met in person					
Q15	I felt I was able to express myself effectively					
Q16	Whenever I made a mistake in SELANGKAH, I could rectify it easily and quickly					
Q17	SELANGKAH gave error messages that clearly instructs me on how to fix the problem					
Q18	I feel comfortable communicating with the clinician using the SELANGKAH.					
Q19	SELANGKAH is an acceptable way to receive healthcare services					
Q20	I think the visits provided over the SELANGKAH system are the same as in-person visits					
Q21	I am satisfied with the SELANGKAH application for home monitoring of COVID-19					
